# Macrophages Mediate Increased CD8 T Cell Inflammation During Weight Loss in Formerly Obese Mice

**DOI:** 10.3389/fendo.2020.00257

**Published:** 2020-04-28

**Authors:** Jayagopi Surendar, Indulekha Karunakaran, Stefan J. Frohberger, Marianne Koschel, Achim Hoerauf, Marc P. Hübner

**Affiliations:** ^1^Institute for Medical Microbiology, Immunology and Parasitology, University Hospital Bonn, Bonn, Germany; ^2^German Center for Infection Research (DZIF), Partner Site Bonn-Cologne, Bonn, Germany

**Keywords:** obesity, inflammation, T cell, macrophages, weight loss, adipose, liver, glucose

## Abstract

Even after successful weight reduction, obese adults tend to quickly regain the lost weight. This raises the question of whether weight loss improves the underlying chronic adipose tissue inflammation characteristic of obesity. In order to improve our understanding of the mechanisms that reshape metabolic organs during weight loss, we investigated the macrophage and T cell function of the liver and adipose tissue on reversing high fat diet (HFD) mice to normal control diet (NCD). Obese mice that were switched to NCD showed an improvement in their metabolic profile that included enhanced glucose and insulin tolerance, decreased cholesterol, triglyceride, serum glutamic-oxaloacetic transaminase (SGOT), and serum glutamic pyruvic transaminase (SGPT) levels that were comparable to NCD controls. However, despite weight loss, increased frequencies, but not total numbers, of IL-17+ and IL-22+ CD4+ T cells, IFN-γ+ and TNF+ CD8+ T cells and IL-17+ and IL-22+ CD8+ T cells were observed in the adipose tissue of mice switched from HFD to NCD compared to NCD and even HFD fed mice. Further, in the liver, IFN-γ+ and TNF+ CD8+ T cell, IL-17+ and IL-22+ CD8+ T cell, macrophage frequencies and their expression of antigen presenting molecules were increased. To determine if macrophages are the major determinants of the sustained inflammation observed during weight loss, we depleted macrophages, which significantly reduced IFN-γ+, TNF+, IL-17+, and IL-22+ CD8+ T cell frequencies in the liver and the adipose tissue. In conclusion, we show that although weight loss improves the metabolic profile, there is an active and ongoing CD8+ T cell inflammation in liver and adipose tissue mediated by macrophages.

## Introduction

Obesity is a major public health problem, which causes the death of at least 2.8 million people every year worldwide. It is causally linked to type 2 diabetes, cardiovascular diseases and cancers (1). In order to achieve weight reduction, various intervention strategies like anti-obesity drugs, bariatric surgery, and lifestyle interventions exist (2). Among these, decreasing caloric intake and physical exercise are by far the most widely adopted methods of choice to promote weight reduction. However, despite successful weight reduction, there is often a quick regain of weight and persistent inflammation in adipose tissue (3, 4). It is therefore important to delineate the underlying mechanisms that contribute to disease risk despite successful weight reduction.

Although it is well-known that obesity is a state of chronic adipose tissue inflammation, little is known about the modulation of this inflammatory state during weight loss. Particularly, the question of how weight loss modulates the specific aspects of obesity-associated inflammation like T cell inflammation is not addressed in detail. A few earlier studies have shown that weight loss did not improve the inflammatory markers' gene expression within the adipose tissue (5). Schmitz et al. (4) have shown that despite improving glucose tolerance, weight loss could not successfully ameliorate adipose tissue inflammation and improve insulin sensitivity. Some reports have also demonstrated an accentuated inflammatory profile after weight loss (6). However, these results are controversial, as other studies have shown that weight loss improves the inflammatory profile of obese subjects (7) and attenuates inflammation in skeletal muscle and liver, but not in adipose tissue (8). Effects may be proportional to the degree of weight loss, as weight loss of 11–16%, but not 5%, resulted in a decrease in adipose tissue inflammation. Furthermore, the increase and persistence of liver and adipose tissue inflammation was also shown to be gender-dependent, with male mice showing a persistent inflammation (9). Of note, weight loss resulted in dynamic effects on macrophages in adipose tissue, with an initial increase followed by a decrease in macrophage numbers (10). Taking into consideration the crucial role of T lymphocytes in obesity related inflammation and the existing literature stating an incomplete resolution of inflammation after weight loss, we investigated the effects of weight loss on type-1 (IFN-γ and TNF) and type-17 (IL-17 and IL-22) cytokines from T cell subsets in the liver and adipose tissue. We report a potent increase in frequencies of cytokine producing CD8 T cells during weight loss from the liver and adipose tissue, which was in part mediated by macrophages.

## Materials and Methods

### Mice

**Figure 1:**
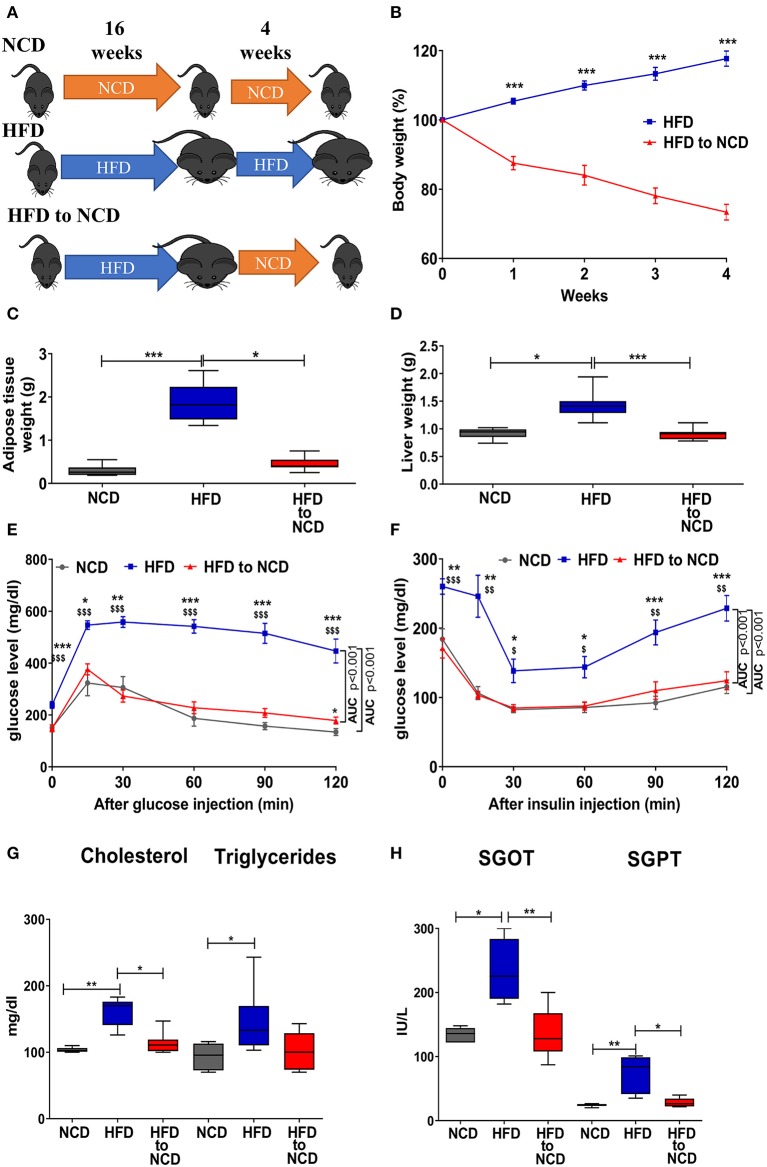
Weight loss in formerly obese mice improves the metabolic profile. **(A)** Schematic experimental design. **(B)** Change in body weight, **(C)** adipose tissue, and **(D)** liver weight 4 weeks after switching from a high fat diet (HFD) to a normal control diet (NCD). **(E,F)** Blood glucose levels over time after i.p. glucose (GTT, **E**) or insulin (ITT, **F**) challenge. *p < 0.05, **p < 0.01, ***p < 0.001 compared to NCD. ^$^*p* < 0.05, ^$$^*p* < 0.01, ^$$$^*p* < 0.001 compared to HFD to NCD. **(G)** Serum lipid profile and **(H)** and liver function enzymes between NCD, HFD and HFD to NCD groups. Pooled data from *n* = 2–3 experiments, 3–5 mice each. Statistical significance was tested by Kruskal-Wallis followed by Dunn's test **(C,D,G,H)** or 2-Way ANOVA followed by Tukey's multiple comparisons test **(B,E,F)**.

All mice were maintained in ventilated cages with a 12-h day/night cycle, food and water *ad libitum*. The experiments were carried out on male C57BL/6J mice purchased from Janvier Labs (Le Genest-St.-Isle, France). Mice were maintained at the animal facilities of the University Hospital Bonn. Starting at 6 weeks of age, mice received either a normal control diet (NCD, 15% fat), a high fat diet (HFD, 60% kilocalories from fat; Research Diets, Inc., Brogaarden, Denmark), or a HFD which was switched after 16 weeks to NCD for an additional 4 weeks (Figure 1A). All mice in the comparative studies were age matched within individual experiments. Animal housing conditions and the procedures were conducted according to European Union animal welfare guidelines. Study protocols were approved by the Landesamt für Natur, Umwelt und Verbraucherschutz, Cologne, Germany (84-02.04.2016.A331).

In a separate experiment, after switching to NCD, macrophages were depleted by intravenous (i.v.) injection of 150 μl of clodronate liposomes (Clodronate Liposomes Foundation; Netherlands; http://clodronate.liposomes.com) and the control mice received equal volumes of PBS liposomes.

### Glucose Tolerance and Insulin Tolerance Test, Lipid Profile and Liver Enzymes

Glucose tolerance tests (GTTs) and insulin tolerance tests (ITTs) were carried out as described elsewhere (11). In brief, 6 h after fasting, mice were intraperitoneally (i.p.) injected with 1 g/kg body weight of glucose solution. At 0, 30, 60, and 120 min blood glucose levels were measured by a glucometer (AccuCheck Advantage; Roche Diagnostics GmbH, Mannheim, Germany).

Four hours after fasting, ITT was performed. Briefly, human insulin (Sanofi-Aventis, Frankfurt, Germany) 1 U of insulin/kg body weight was i.p. injected and at 0, 30, 60, and 120 min blood glucose levels were measured. The area under the curve (AUC) was derived by calculating the area between the x-axis and a given curve using GraphPad Prism software (version 8.3; GraphPad Software, San Diego, Calif., USA).

Lipid profiles and liver enzymes—serum glutamic-oxaloacetic transaminase (SGOT) and serum glutamic pyruvic transaminase (SGPT)—were measured using Reflotron (Roche Diagnostics GmbH) according to the manufacturer's protocol.

### Isolation of Stromal Vascular Fraction From Adipose Tissue and Leucocytes From Liver

Mice were deeply anesthetized by i.p. injection of 10 mg/kg xylazin (Rompun® Bayer, Germany) + 100 mg/ml ketamine (Ratiopharm GmbH Germany). Mice were intracardially perfused with 1x PBS for 5 min to remove circulating and non-adhered blood leukocytes from the organs (12).

After perfusion, adipose tissue stromal vascular fraction (SVF) and liver lymphocytes were isolated. In brief, the excised epididymal adipose tissue from the mice was digested with 0.2 mg/ml of collagenase (Sigma-Aldrich; Taufkirchen, Germany) in DMEM medium at 37°C for 40 min. After the digestion, the adipocytes were removed and SVF pellet was filtered by passing through a 40 μm filter after red blood cell lysis (Invitrogen, Thermo Fisher Scientific; Carlsbad, CA, USA).

To isolate cells from the liver, the liver was minced into small pieces followed by digestion with 0.5 mg/ml collagenase A (Roche, Basel, Switzerland) at 37°C for 30 min. After the digestion single cell suspension was generated by passing the digested tissue through a 70 μm filter. Lymphocytes were enriched from the homogenate using a percoll gradient.

### Cell Culture

After cell enumeration from SVF and liver single cell suspension, isolated cells were cultured in 12-well tissue culture at concentrations of 1 × 10^6^ cells/ml in the presence of phorbol myristate acetate (PMA) (50 ng/ml) and ionomycin (1 μg/ml) for 6 h in RPMI-1640 medium (Gibco, Thermo Fischer scientific) at 37°C. After 2 h, Golgi Stop/Golgi Plug (BD Biosciences, Heidelberg, Germany) was added and cells were harvested 4 h later.

### Flow Cytometry

After *in vitro* stimulation, cells were harvested and incubated in fixation/permeabilization buffer overnight (eBiosciences; Darmstadt, Germany). Next, cells were blocked with PBS/1% BSA including 0.1% rat IgG for 30 min (Sigma-Aldrich). After a washing step, the cells were incubated with permeabilization buffer (eBioscience) for an additional 20 min. After washing, cells were stained for flow cytometry with antibodies against CD4-PE/Cy7, CD8-PerCP/Cy5.5 and intracellular cytokines IFN-γ-PE, TNF-FITC or IL-17A-PE, IL-22-APC. For quantification of regulatory T cells, cells were permeabilized and stained with CD4-PE/Cy7 and FoxP3-FITC for 30 min. For identification of macrophages, the whole cell population was selected and doublet cells were excluded by FSC-W and SSC-A characteristics followed by gating of F4/80-PerCP/Cy5.5+ and CD11b-APC/Cy7+ cells as macrophages. Macrophages were shown as percentage of total SVF cells. All antibodies were purchased from eBioscience (Darmstadt, Germany) or Biolegend (Fell, Germany). Data were acquired with a BD FACS Canto System (BD Biosciences) and analyzed using FlowJo 10 (Flowjo LLC; Ashland, Oregon) software. During analysis, gates were set using the FMO (fluorescence minus one) approach.

### Real Time PCR

From adipose tissue RNA was extracted using the RNeasy mini kit (Qiagen). RNA was reverse transcribed with the Omniscript RT Kit (Qiagen) according to the manufacturer's instructions with oligo-d(T) primers (Roche; Penzberg, Germany). Real-time PCR was performed with the Thermo Fisher QuantStudio 5 using the TaqMan universal PCR master mix (Thermo Fisher Scientific). TaqMan probes for arginase-1 (*Arg-1*), resistin-like molecule alpha (*RELM-*α) and *Tnf* were analyzed and hypoxanthine-guanine phosphoribosyltransferase (*hprt*) was used as an endogenous control (Thermo Fisher Scientific). The relative CT (threshold cycle at the exponential phase of amplification) method was used to calculate the qPCR results. Delta CT was calculated as CT (gene of interest)—CT (*hprt*). The fold change was calculated as 2^−ΔCT^.

### Statistics

GraphPad Prism software version 8.3 was used for statistical analyses (GraphPad Software, San Diego, CA, USA). Data were analyzed for statistical significance by Kruskal Wallis followed by Dunn's test for multiple comparisons and Mann Whitney *U*-test for comparison of two groups. Changes in GTT and ITT as well as body weight over time were analyzed by 2-Way ANOVA followed by Tukey's multiple comparisons test. *P* < 0.05 were considered statistically significant.

## Results

### Weight Loss Improves Insulin Sensitivity, Lipid Profile and Liver Enzymes

Mice were fed a high fat diet (HFD) for a period of 16 weeks to induce weight gain and were then maintained on HFD or switched to normal control diet (NCD) for 4 weeks to induce body weight reduction (Figure 1A). HFD to NCD switching (formerly obese mice) resulted in a significant reduction in body weight (Figure 1B), adipose tissue weight (Figure 1C), and liver weight (Figure 1D).

After 20 weeks of HFD, impaired glucose and insulin tolerance were observed, whereas formerly obese mice that were switched to a NCD for the last 4 weeks had a glucose and insulin tolerance that was comparable to NCD controls (Figures 1E,F). Further, circulating levels of cholesterol (Figure 1G), triglycerides (Figure 1G) and the liver enzymes SGOT (Figure 1H) and SGPT (Figure 1H) were significantly increased in HFD fed mice compared to NCD control mice and formerly obese mice.

### Increased Relative Numbers of Pro-inflammatory Cytokine-Producing T Cells in Adipose Tissue and Liver Are Sustained Despite Weight Loss

**Figure 2:**
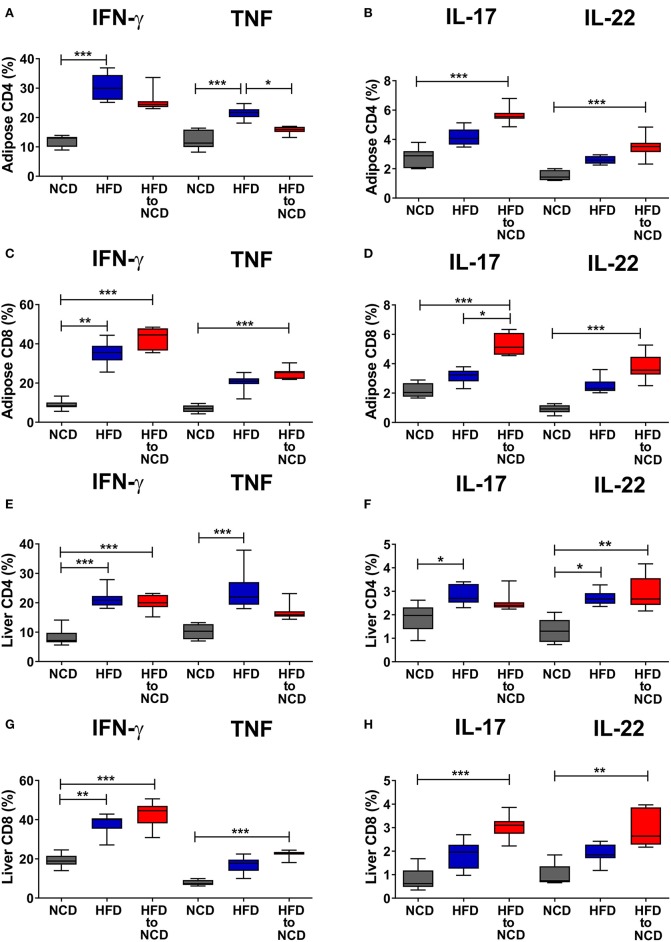
Switching obese mice to a normal control diet increases frequencies of pro-inflammatory cytokine producing CD8+ T cells in adipose tissue and liver. Animals were fed for 20 weeks on a high fat diet (HFD), normal control diet (NCD) or were switched after 16 weeks of a HFD to a NCD for 4 weeks. Frequency of IFN-γ+ and TNF+ **(A,C)**, IL-17+ and IL-22+ **(B,D)** CD4+ T cells **(A,B)**, and CD8+ T cells **(C,D)** within the adipose tissue. Frequency of IFN-γ+ and TNF+ **(E,G)**, IL-17+ and IL-22+ **(F,H)** CD4+ T cells **(E,F)**, and CD8+ T cells **(G,H)** within the liver. Cytokine expression of T cells was determined following PMA/Ionomycin stimulation. *p < 0.05, **p < 0.01, ***p < 0.001. Pooled data from *n* = 2–3 experiments with 3–5 mice each. Statistical significance was tested by Kruskal-Wallis followed by Dunn's test.

We next investigated the effect of weight loss on frequencies of type-1 and type-17 cytokine positive CD4+ and CD8+ T cells in the liver and adipose tissue. The gating strategy for T cells is shown in Supplementary Figure 1. In the adipose tissue, switching from HFD to NCD resulted in decreased frequencies of IFN-γ+ (*p* > 0.05) and TNF+ CD4+ T cells (p<0.05) compared to HFD controls (Figure 2A). On the contrary, frequencies of IL-17+ and IL-22+ CD4+ T cells (Figure 2B), IFN-γ+ and TNF+ CD8+ T cells (Figure 2C) and IL-17+ and IL-22+ CD8+ T cells (Figure 2D) were highest in the HFD to NCD group. In the liver, frequencies of TNF+ CD4+ and IL-17+ CD4+ T cells were found to be decreased (Figures 2E,F), whereas the percentages of IFN-γ+ and TNF+ CD8+ T cells (Figure 2G) and IL-17+ and IL-22+ CD8+ cells (Figure 2H) were highest in the formerly obese group. With regard to total numbers, HFD significantly increased the number of CD4+ type-17 (IL-17 and IL-22) (Supplementary Figure 2B), CD8+ type-1 (IFN-γ+ and TNF) (Supplementary Figure 2C), CD8+ type-17 (Supplementary Figure 2D) and by trend CD4+ type-1 (Supplementary Figure 2A) cytokine positive cells compared to the NCD group in adipose tissue. Reversal of the diet from HFD to NCD significantly reduced IFN-γ+ CD8+ T cell (Supplementary Figure 2C) and IL-17+ CD8+ cell numbers (Supplementary Figure 2D) in adipose tissue. Total numbers of liver CD4+ type-1 and type-17 cytokine positive T cells increased in the HFD group compared to the NCD group and switching diet from HFD to NCD significantly reduced CD4+ type-1 and type-17 cytokine positive T cells compared to the HFD group (Supplementary Figures 2E,F). HFD further increased liver CD8+ type-1 and type-17 cytokine positive T cells compared to the NCD group (Supplementary Figures 2G,H). Frequencies of FoxP3+ regulatory T cells were significantly reduced after HFD in both adipose tissue and liver and remained low after the reversal of the diet (Supplementary Figures 3A,B).

### Macrophage Depletion Attenuates CD8+ T Cell Cytokine Production in Adipose Tissue and Liver

**Figure 3:**
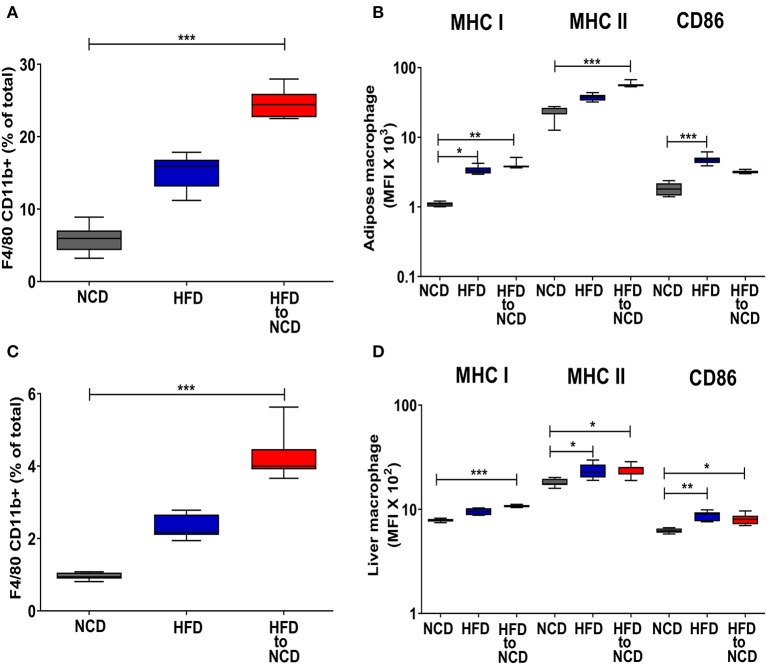
Switching obese mice to a normal control diet leads to an increased frequency of adipose tissue and liver macrophages and increased expression of antigen presenting molecules. Animals were fed for 20 weeks on a high fat diet (HFD), normal control diet (NCD) or were switched after 16 weeks of a HFD to a NCD for 4 weeks. **(A)** Frequency of macrophages in the visceral adipose tissue and **(B)** mean fluorescence intensity (MFI) of MHCI, MHCII and CD86 on the adipose tissue macrophages. **(C)** Frequency of macrophages in liver and **(D)** MFI of MHCI, MHCII and CD86 on the liver macrophages. *p < 0.05, **p < 0.01, ***p < 0.001. Pooled data from *n* = 2–3 experiments with 3–5 mice each. Statistical significance was tested by Kruskal-Wallis followed by Dunn's test. NCD, normal control diet; HFD, high fat diet, HFD to NCD–HFD switched to NCD.

Intriguingly, the frequencies of CD11b+ adipose tissue macrophages (Figure 3A) and the expression of MHCI and MHCII on adipose tissue macrophages were highest in HFD animals that were switched to a NCD (Figure 3B). A similar pattern on macrophages was observed in the liver as shown by increased liver macrophage frequencies (Figure 3C) and the elevated expression of MHCI on liver macrophages (Figure 3D). The total number of macrophages in adipose tissue and liver were highest in the animals that were maintained on a HFD and remained significantly increased following reversal of the diet in comparison to the NCD group (Supplementary Figures 4A,B). The macrophage gating strategy is shown in Supplementary Figure 5. The expression of the alternatively activated macrophage markers Arginase-1 and RELM-α were reduced in the adipose tissue of the HFD group in comparison to the NCD group (*p* > 0.05) and tended to increase after the reversal of the diet (*p* > 0.05; Supplementary Figures 6A,B). HFD increased the TNF expression in adipose tissue compared to the NCD group and switching the diet reduced the TNF expression (Supplementary Figure 6C). Collectively, there was no clear increase of alternatively activated macrophage-associated gene expression in adipose tissue macrophages 4 weeks after the reversal of diet.

**Figure 4:**
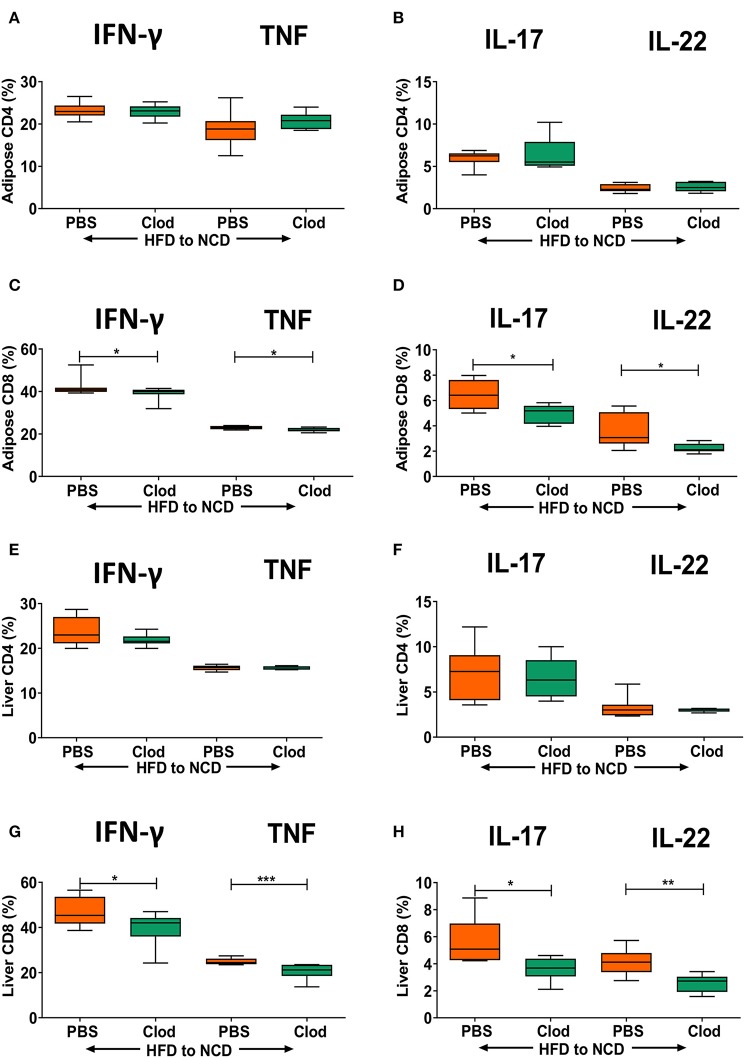
Depletion of macrophages reduces frequencies of pro-inflammatory cytokine-producing CD8+ T cells in formerly obese mice. Animals were fed for 16 weeks on a HFD and switched to a NCD for 4 weeks, macrophages were depleted by clodronate liposomes (Clod) injection immediately after switching to the NCD diet. Controls receiving the same switch from a HFD to a NCD were treated with PBS liposomes (PBS). **(A–D)** Frequency of IFN-γ+ and TNF+ **(A,C)**, IL-17+ and IL-22+ **(B,D)** CD4+ T cells **(A,B)** and CD8+T cells **(C,D)** in adipose tissue. **(E–H)** Frequency of IFN-γ+ and TNF+ **(E,G)**, IL-17+ and IL-22+ **(F,H)** CD4+ T cells **(E,F)** and CD8+T cells **(G,H)** in liver. *p < 0.05, **p < 0.01, ***p < 0.001. Pooled data from *n* = 2 experiments, 3–5 mice each. Mann Whitney *U*-test was used to assess the statistical significant differences.

As we observed that frequencies of T cell cytokine positive cells are in parallel with macrophage accumulation and their expression of antigen presenting molecules in adipose tissue and liver, we set out to determine if macrophages are a major determinant of sustained inflammation observed during weight loss. Macrophage depletion was confirmed by flow cytometry (Supplementary Figure 5), but did not affect frequencies of CD4+ cytokine positive cells (Figures 4A,B), but significantly diminished the frequency of IFN-γ+, TNF+ (Figure 4C) as well as IL-17+ and IL-22+ CD8+ T cells (Figure 4D) in the adipose tissue. Similar to adipose tissue, depletion of macrophages had no effect on frequencies of IFN-γ+, TNF+ (Figure 4E) as well as IL-17+ and IL-22+ CD4+ T cells (Figure 4F) in the liver. However, frequencies of IFN-γ+, TNF+ (Figure 4G) and IL-17+ and IL-22+ CD8+ T cells (Figure 4H) in the liver were significantly decreased upon macrophage depletion compared to the control group. Macrophage depletion did not affect any of the metabolic parameters such as body weight (Supplementary Figure 7A), adipose (Supplementary Figure 7B), and liver weight (Supplementary Figure 7C), GTT (Supplementary Figures 7D,E), and ITT (Supplementary Figures 7F,G), lipid profiles (Supplementary Figures 7H,I), and liver enzymes (Supplementary Figures 7J,K).

## Discussion

A combination of physical exercise and dietary changes is considered as the most effective non-invasive intervention strategy for obesity. However, weight loss programs have been shown to bring about only an average weight reduction of 10% and despite the weight loss, the majority of patients quickly regain the lost weight (13). This leads to the question of whether weight loss improves the adverse metabolic and inflammatory profile underlying obese conditions. In order to better understand the biological mechanisms that reshape metabolic organs during weight loss, we investigated the macrophage and T cell function of the liver and adipose tissue on reversing HFD mice to NCD. Although an improvement in insulin sensitivity, lipid profile, and liver enzymes were evident after weight loss, the adipose tissue, and liver macrophage frequencies were increased and the expression of antigen presenting molecules on the macrophages were also increased. Total macrophage numbers increased in the metabolic organs during HFD and remained significantly increased following the reversal of the diet. Depletion of macrophages was sufficient to specifically limit the specific type 1 and type 17 CD8+ T cell frequencies, but not frequencies of type 1 and type 17 positive CD4+ T cells, from the adipose tissue and liver. Our findings are in agreement with the results of Zamarron et al. (5), who showed increased infiltration of macrophages in the adipose tissue up to 6 months after diet intervention. Further, Fischer et al. (14) have shown the presence of an inflammatory imprint in liver and perigonadal fat even after normalization of the metabolic parameters. Taken together these results argue in favor of liver and adipose tissue intrinsic memory of previous obesity. However, there are other reports that have shown that weight loss improves the inflammatory profile of obese subjects (7, 8).

When we probed into T cell alterations in our study, HFD increased type 1 cytokine positive CD4+ T cell frequencies in metabolic organs and switching HFD mice to NCD reduced them. In contrast to our findings, Zamarron et al. showed that HFD reduces frequencies of IFN-γ+ CD4+ T cells in adipose tissue and this phenotype was reversed after switching to a NCD (5). Regarding type-17 cells, reversing HFD to NCD increased the frequency of IL-17+ and IL-22+ CD4+ T cells in the adipose tissue and IL-22+ CD4+ T cells in the liver. Nonetheless, macrophage depletion did not rescue this effect of the reversed diet. Therefore, further studies are needed to identify the factors that regulate Th17 cells in the metabolic organs during weight loss. Intriguingly, the frequencies, but not total numbers, of type 1 and type 17 cytokine positive CD8+ T cells increased after weight loss. As increased macrophage accumulation was accompanied by increased frequencies of cytokine positive T cells in liver and adipose tissue, we depleted macrophages, which significantly reduced the frequencies of IFN-γ+, TNF+, IL-17+, and IL-22+ positive CD8+ T cells of the liver and the adipose tissue. It was previously shown that formerly obese mice regain body weight much faster than constantly lean counterparts (6). CD4+ T cells could be essentially involved as H2A^−/−^ mice, which lack CD4+ T cells, did not develop an obesity memory and an accelerated weight regain occurred when CD4+ T cells of formerly obese mice were introduced to Rag1^−/−^ mice (6). However, we did not see a significant impact of macrophages depletion on systemic parameters in obese mice. In contrast to our finding, Feng et al. showed that macrophage depletion modulates systemic metabolic parameters even in lean mice (15). We speculate that the reason for the lack of effect on metabolism in our study could be the shorter follow up period of 4 weeks after macrophage depletion and/or type of diet composition. There were slight but non-significant reductions in body weight, adipose tissue weight, and SGPT levels upon macrophage depletion. Thus, a longer follow up time might have resulted in a significant improvement of metabolic parameters.

One limitation of this study is that we have not validated our findings in humans and further studies are needed to investigate the impact of macrophages on adipose tissue inflammation and systemic metabolic parameters during weight loss in humans. It is not clear how and why weight loss leads to pro-inflammatory T cell responses. It is possible that the inflammation might have some beneficial effects as a low level of inflammation has been shown to be a pre-requisite for metabolic homeostasis (16) and the increased adipose tissue macrophages could help in resolving the excess deposition of extracellular matrix (17).

In conclusion, we show that although weight loss improves the metabolic profile, there is a robust increase in macrophage frequencies and antigen presentation accompanied by an active and ongoing/augmented CD8+ T cell inflammation both in liver and adipose tissue for at least 4 weeks after stopping a HFD. Depletion of macrophages reduced the expression of CD8+ T cell cytokines, suggesting that macrophages are major mediators of CD8+ T cell inflammation during weight loss. Therefore, it is possible that the sustained, increased CD8+ T cell inflammation in liver and adipose tissue could be the reason for the quick regaining of body weight.

## Data Availability Statement

All datasets generated for this study are included in the article/Supplementary Material.

## Ethics Statement

The animal study was reviewed and approved by the Landesamt für Natur, Umwelt und Verbraucherschutz, Cologne, Germany (84-02.04.2016.A331).

## Author Contributions

JS and MH: conceptualization and project administration. JS and SF: formal analysis. JS, IK, SF, and MK: methodology. AH and MH: resources and supervision. JS, IK, and MH: writing—original draft. JS, IK, SF, AH, and MH: writing—review and editing.

## Conflict of Interest

The authors declare that the research was conducted in the absence of any commercial or financial relationships that could be construed as a potential conflict of interest.
