# Supplementary material for: Macrophages Mediate Increased CD8 T Cell Inflammation During Weight Loss in Formerly Obese Mice
Source: Front Endocrinol (Lausanne). 2020 Apr 28;11:257. doi: 10.3389/fendo.2020.00257 (PMC7198814; doi:10.3389/fendo.2020.00257)
Supplement: Supplementary file 1 [file Data_Sheet_1.PDF]

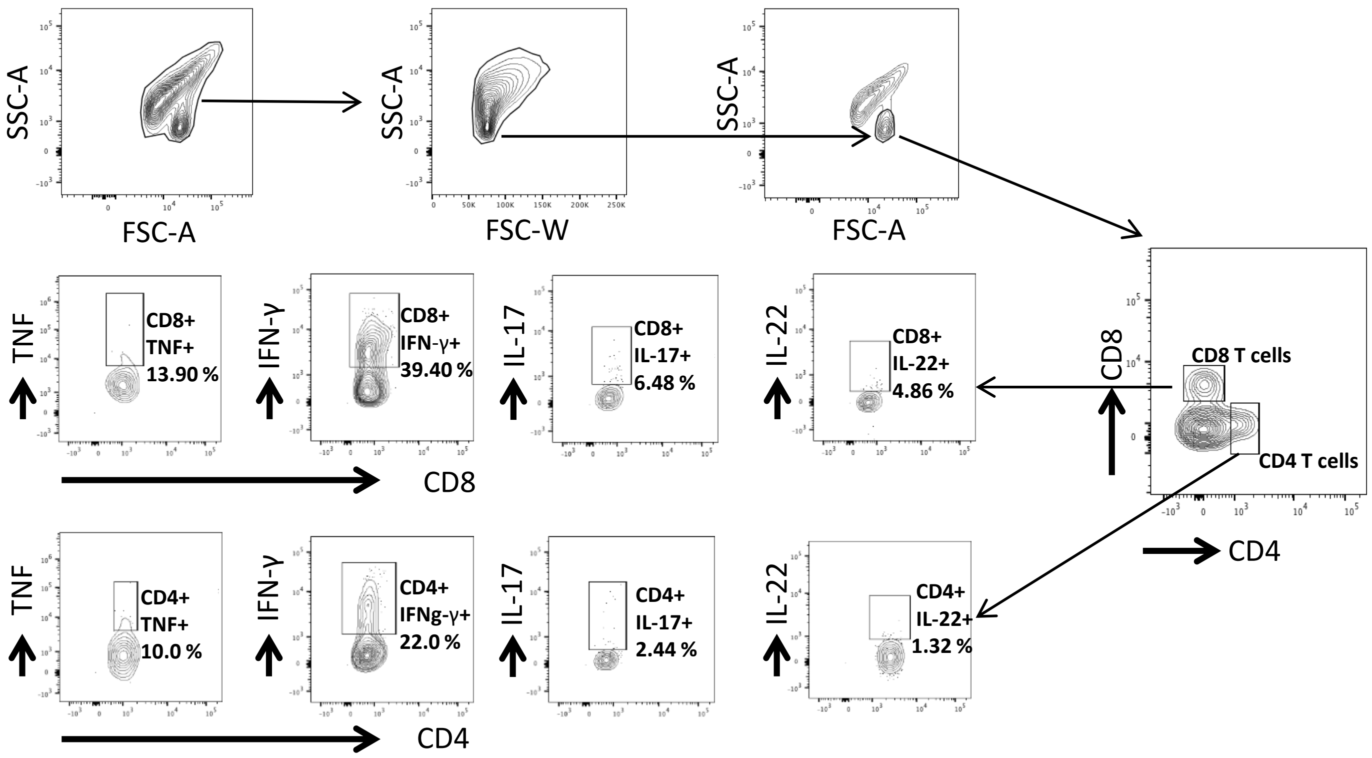

Supplementary Figure 1. Gating strategy for cytokine-producing CD4+ and CD8+ T cells. IFN-γ, TNF, IL-17, and IL-22 cytokine producing CD4+ and CD8+ T cells within the adipose stromal vascular fraction and liver were determined after in vitro PMA/Ionomycin stimulation by flow cytometry. The complete cell population was selected and doublets were excluded by FSC-W characteristics. Based on FSC-A and SSC-A, lymphocytes were selected and T cells were identified based on CD4 and CD8 positivity. Using the fluorescence minus one approach, intracellular expression of IFN-γ, TNF, IL-17 and IL-22 were gated from CD4+ and CD8+ cells.
