# Supplementary material for: Macrophages Mediate Increased CD8 T Cell Inflammation During Weight Loss in Formerly Obese Mice
Source: Front Endocrinol (Lausanne). 2020 Apr 28;11:257. doi: 10.3389/fendo.2020.00257 (PMC7198814; doi:10.3389/fendo.2020.00257)
Supplement: Supplementary file 2 [file Data_Sheet_2.PDF]

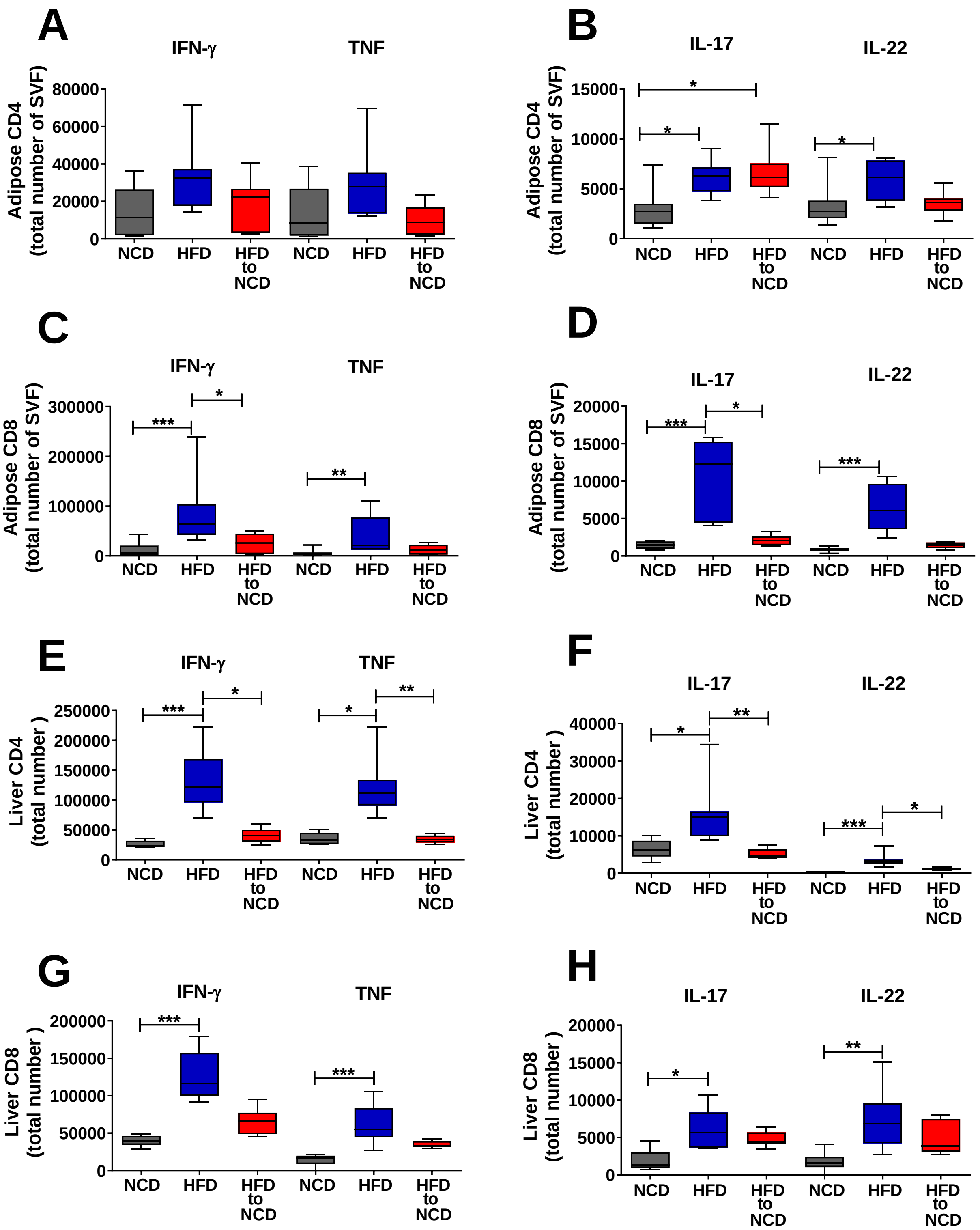

Supplementary Figure 2: HFD increases total type-1 and type-17 cytokine positive T cell numbers in adipose tissue and liver and this is partially reduced by reversal of the diet. Animals were fed for 20 weeks on a high fat diet (HFD), normal control diet (NCD) or were switched after 16 weeks of a HFD to a NCD for 4 weeks. Total number of IFN-γ+ and TNF+ **(A, C)**, IL-17+ and IL-22+ **(B, D)** CD4+ T cells and CD8+ T cells within the adipose tissue. Total number of IFN-γ+ and TNF+ **(E, G)**, IL-17+ and IL-22+ **(F, H)** CD4+ T cells and CD8+ T cells within the liver. Pooled data from n=2-3 experiments with 3-5 mice each. Statistical significance was tested by Kruskal-Wallis followed by Dunn's test. *p<0.05, **p<0.01, ***p<0.001.
