# Supplementary material for: Macrophages Mediate Increased CD8 T Cell Inflammation During Weight Loss in Formerly Obese Mice
Source: Front Endocrinol (Lausanne). 2020 Apr 28;11:257. doi: 10.3389/fendo.2020.00257 (PMC7198814; doi:10.3389/fendo.2020.00257)
Supplement: Supplementary file 3 [file Data_Sheet_3.PDF]

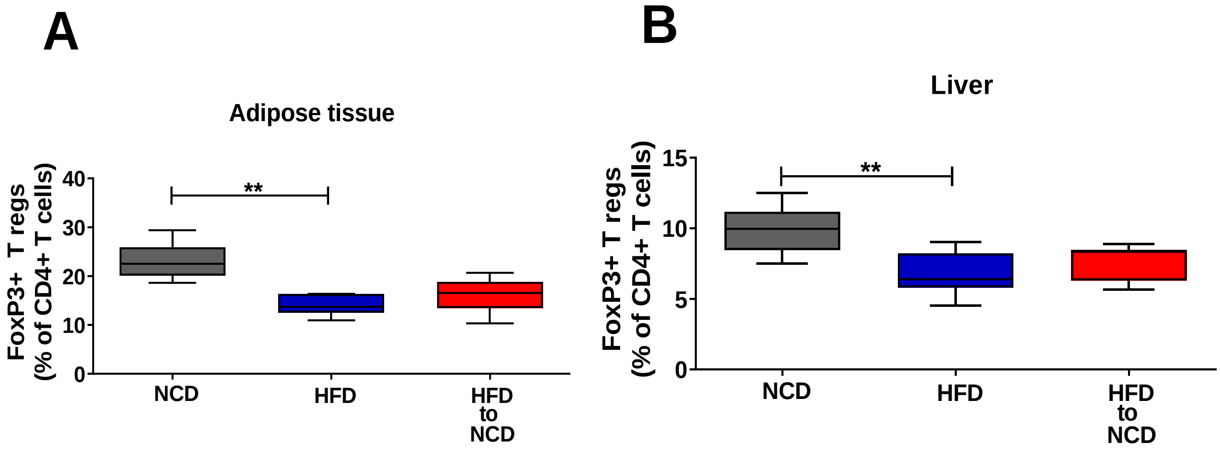

Supplementary Figure 3. Switching obese mice to a normal control diet does not lead to an increase in regulatory T cells in adipose tissue and liver. Frequencies of adipose tissue **(A)** and liver **(B)** CD4+ T cells expressing FoxP3+ in animals that were fed for 20 weeks on a high fat diet (HFD), normal control diet (NCD) or were switched after 16 weeks of a HFD to a NCD for 4 weeks. Pooled data from n=2 experiments with 4-5 mice each. Statistical significance was tested by Kruskal-Wallis followed by Dunn's test. **p<0.01.
