# Supplementary material for: Macrophages Mediate Increased CD8 T Cell Inflammation During Weight Loss in Formerly Obese Mice
Source: Front Endocrinol (Lausanne). 2020 Apr 28;11:257. doi: 10.3389/fendo.2020.00257 (PMC7198814; doi:10.3389/fendo.2020.00257)
Supplement: Supplementary file 6 [file Data_Sheet_6.PDF]

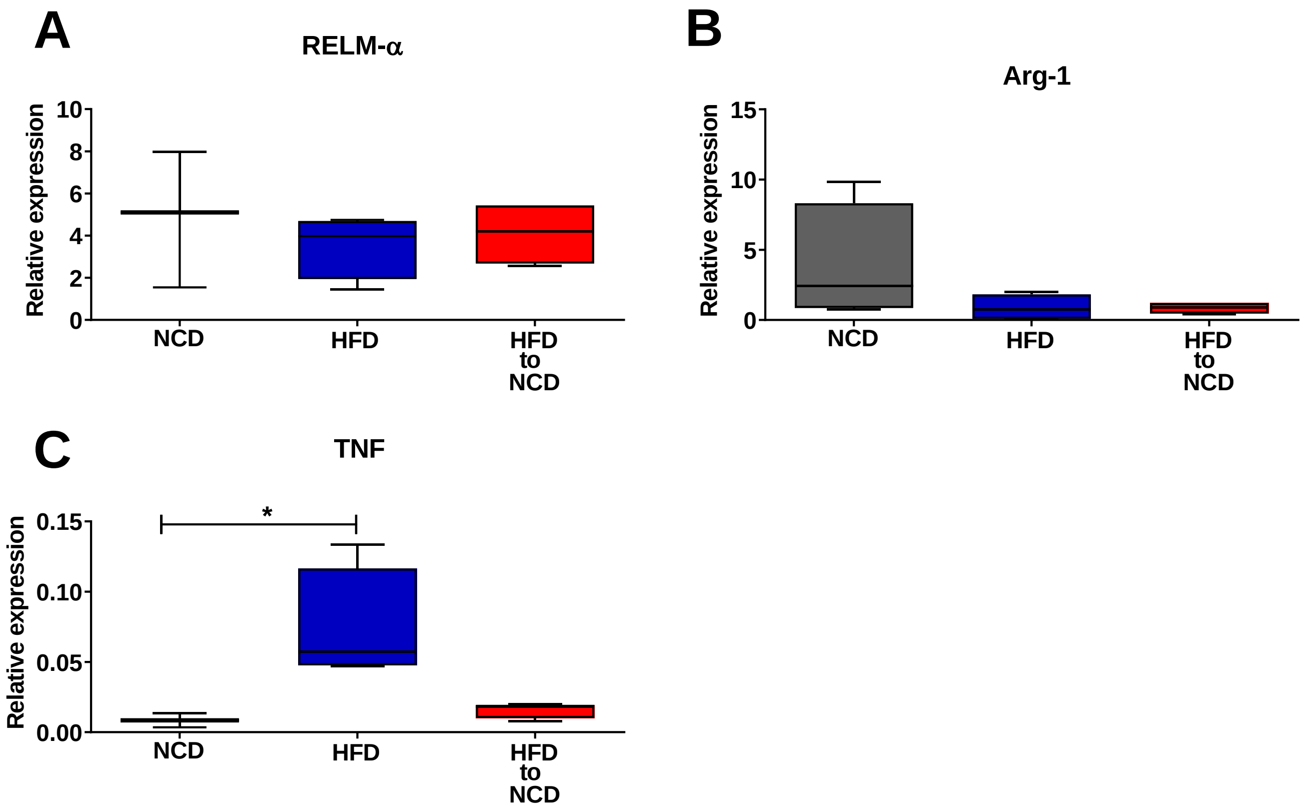

Supplementary Figure 6. HFD modulates macrophage specific gene expression in adipose tissue tissue. Relative messenger RNA expression of arginase-1 (Arg-1) **(A)**, RELM-α **(B)** and TNF **(C)** in adipose tissue of animals that were fed for 20 weeks on a high fat diet (HFD), normal control diet (NCD) or were switched after 16 weeks of a HFD to a NCD for 4 weeks. Data from one experiment with 4 mice. Statistical significance was tested by Kruskal-Wallis followed by Dunn’s test. *$p<0.05$.
