# Supplementary material for: Macrophages Mediate Increased CD8 T Cell Inflammation During Weight Loss in Formerly Obese Mice
Source: Front Endocrinol (Lausanne). 2020 Apr 28;11:257. doi: 10.3389/fendo.2020.00257 (PMC7198814; doi:10.3389/fendo.2020.00257)
Supplement: Supplementary file 7 [file Data_Sheet_7.PDF]

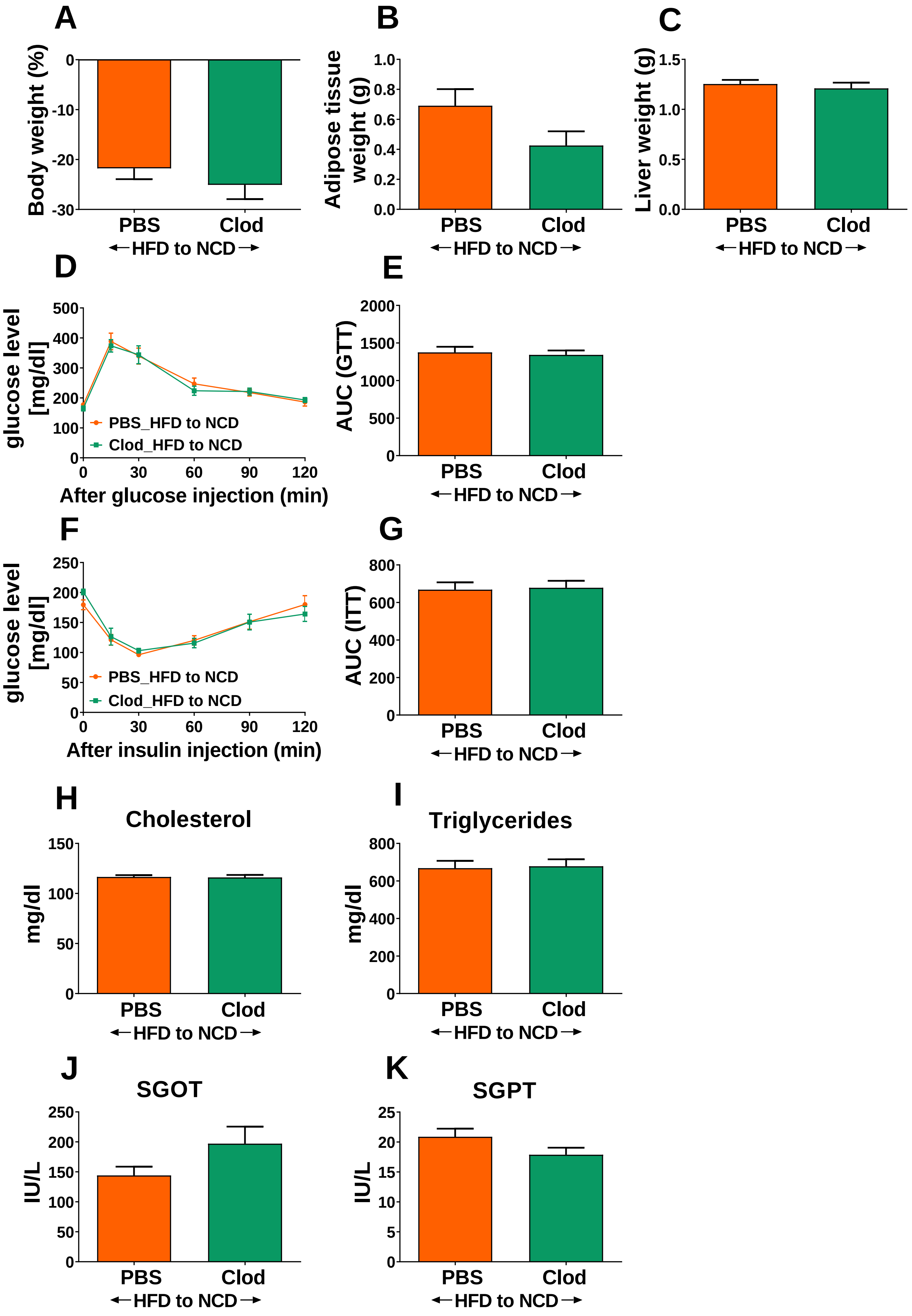

Supplementary Figure 7: Depletion of macrophages does not affect the metabolic profile in formerly obese mice. Animals were fed for 16 weeks on a HFD and switched to a NCD for 4 weeks. **(A)** Percent changes in body weight, **(B)** adipose tissue and **(C)** liver weight between clodronate liposomes (Clod) treated and control-liposomes (PBS) treated HFD to NCD mice. **(D-G)** Blood glucose levels over time after i.p. glucose (GTT) or insulin (ITT) challenge. Blood glucose levels over time **(D, F)** and area under the curve of the GTT (**E**) and ITT **(G)** challenge. **(H)** Serum cholesterol, **(I)** triglyceride, **(J)** SGOT, and **(K)** SGPT levels in control and clodronate treated mice. Pooled data from n=2 experiments with 3-5 mice each. Statistical significance was analysed by Mann Whitney U test **(A-C, E, G-K)** or 2-Way ANOVA followed by Tukey's multiple comparisons test **(D, F)**.
